# *In Vitro* Colonization of the Muscle Extracellular Matrix Components by *Escherichia coli* O157:H7: The Influence of Growth Medium, Temperature and pH on Initial Adhesion and Induction of Biofilm Formation by Collagens I and III

**DOI:** 10.1371/journal.pone.0059386

**Published:** 2013-03-13

**Authors:** Caroline Chagnot, Allison Agus, Sandra Renier, Frédéric Peyrin, Régine Talon, Thierry Astruc, Mickaël Desvaux

**Affiliations:** 1 INRA, UR454 Microbiologie, Clermont-Ferrand, France; 2 INRA, UR370 Qualité des Produits Animaux, Clermont-Ferrand, France; Ghent University, Belgium

## Abstract

Enterohemorrhagic *Escherichia coli* (EHEC) O157:H7 are responsible for repeated food-poisoning cases often caused by contaminated burgers. EHEC infection is predominantly a pediatric illness, which can lead to life-threatening diseases. Ruminants are the main natural reservoir for EHEC and food contamination almost always originates from faecal contamination. In beef meat products, primary bacterial contamination occurs at the dehiding stage of slaughtering. The extracellular matrix (ECM) is the most exposed part of the skeletal muscles in beef carcasses. Investigating the adhesion to the main muscle fibrous ECM proteins, insoluble fibronectin, collagen I, III and IV, laminin-α2 and elastin, results demonstrated that the preceding growth conditions had a great influence on subsequent bacterial attachment. In the tested experimental conditions, maximal adhesion to fibril-forming collagens I or III occurred at 25°C and pH 7. Once initially adhered, exposure to lower temperatures, as applied to meat during cutting and storage, or acidification, as in the course of post-mortem physiological modifications of muscle, had no effect on detachment, except at pH_u_. In addition, dense biofilm formation occurred on immobilized collagen I or III and was induced in growth medium supplemented with collagen I in solution. From this first comprehensive investigation of EHEC adhesion to ECM proteins with respect to muscle biology and meat processing, new research directions for the development of innovative practices to minimize the risk of meat contamination are further discussed.

## Introduction

Worldwide, the occurrence of food poisoning following the consumption of products contaminated with enterohemorrhagic *Escherichia coli* (EHEC) is recurrent [1]. EHEC outbreaks and sporadic cases incriminate some meat, milk or vegetable products as well as water-based drinks [2], [3]. Human infections, though, often follow the consumption of beef meat, especially minced meat, and often involve strains of the serotype O157:H7. The EHEC reference strain O157:H7 EDL933 was originally isolated from contaminated burgers (beef patty) responsible for an outbreak of hemorrhagic colitis that occurred in the United States of America [4]. From this episode in 1982, the significance of EHEC as a serious public health problem was first recognized. EHEC infection manifests clinically with diarrhea and abdominal cramps before proceeding to hemorrhagic colitis characterized by bloody diarrhea [5], [6]. Although it can occur at any age, EHEC infection is predominantly a pediatric illness [7]. It can cause into most acute forms of life-threatening complications, namely haemolytic uraemic syndrome (HUS) especially in young children less than 5 years old, or more rarely into thrombotic thrombocytopenic purpura (TTP) [8], [9].

Ultimately, EHEC contamination of food products originates primarily from faecal contamination. Ruminants such as bovines are the main natural reservoir for EHEC [1]. In the case of beef, hygienic slaughtering practices reduce faecal contamination of carcasses (prevention of evisceration accidents, cross contamination and poor hygiene) but cannot guarantee the absence of *E. coli* O157:H7 from meat [10]. Primary bacterial contamination occurs often inevitably at the dehiding stage of processing where bacteria can be transferred from hides to beef carcasses. Post animal slaughther, the muscle converts to meat undergoing a succession of post-mortem physiological changes [11]. According to the European Community (EC) regulation specifying the hygiene rules for foodstuffs (n°853/2004), animal slaughter and cutting of carcases into quarters can be carried out at room temperature. However, during further cutting, storage and/or transport, meat must reach and be maintained at 7°C. From then on the temperature of meat preparations and minced meat must not exceed 4°C and 2°C respectively. While molecular mechanisms of EHEC pathogenicity have been subjected to intense research [12], [13], the molecular aspects of food contamination clearly lags behind. Such basic knowledge is of great importance to allow for further improvements to quantitative risk assessment in the management of *E. coli* O157:H7 in the beef processing industry [14].

Attachment of *E. coli* O157:H7 to beef meat has been reported [15], [16], [17], [18] and identified between muscle fibres within the connective tissue [19], [20]. However, the molecular interactions between the bacterial cells and the muscle extracellular matrix (ECM) components are more controversial. ECM is composed of two main classes of macromolecules, the fibrous proteins and the proteoglycans [21]. In skeletal muscle tissue, fibrous proteins are the predominant components of the ECM, essentially comprised of collagens I, III and IV, insoluble fibronectin (i-fibronectin), laminin-α2 and elastin [22], [23], [24], [25]. Using a surface plasmon resonance biosensor, binding of collagen I and laminin to the cell surface of *E. coli* O157:H7 was reported [20], [26], [27]. The role of pili in mediating the attachment of EHEC cells to meat was suggested but not ascertained and other cell surface determinants related to the virulence may also be involved [15], [26], [28]. Importantly, the expression of virulence factors in EHEC is inducible and depends on growth conditions [29], [30]. Surprisingly enough, a most recent investigation could not provide evidence of significant attachment of O157:H7 EHEC strains to any of the tested immobilized ECM proteins [31]. Altogether, this prompted us to reinvestigate the adhesion of EHEC O157:H7 to the main ECM fibrous proteins present in beef meat.

## Materials and Methods

### Bacterial strains and culture conditions

The non-toxigenic isogenic mutant of EHEC O157:H7 EDL933, deleted of the *stx1* and *stx2* genes [32], [33], were used in this study. Bacteria were cultured in different nutrient media either chemically defined, i.e. DMEM (Dulbecco's modified eagle medium, Gibco), M9 [34] and MinCa (minimal casein) media [35], or complex undefined, i.e. LB (lysogeny broth) [36], TSB (tryptic soy broth, Becton-Dickinson), BHI (brain-heart infusion, Becton-Dickinson), LH (Leedle Hespell) [37], BCC (bovine caecum content) and BJIC (bovine jejunum-ileum content) sterile media [38]. Contents of different parts of the bovine digestive tract were collected as previously described to prepare LH, BCC and BJIC [38]. From −80°C stock culture previously grown in the respective medium, strains were plated on the relevant agar medium and incubated overnight at 39°C (bovine temperature) [38]. A preculture was set up from one bacterial colony grown in the respective nutrient broth medium at 39°C in an orbital shaker at low speed (70 rpm) till stationary phase.

Fluorescent bacterial strains were obtained following transformation with the vector pSARE-Red1 expressing the fluorescent protein mRuby [39]. Briefly, the gene encoding mRuby was amplified by PCR with high-fidelity TaKaRa LA Taq DNA polymerase from pmRuby using mRubF (TTATATCATGAACAGCCTGATCAAAGAAAACATGCGG) and mRubR (TTATACTCGAGGCATGCTTACCCTCCGCCCAGGC) primers. The amplicon was cloned into pIMK2 [40] following DNA digest with NcoI/XhoI restriction enzymes, ligation and electroporation into *E. coli* TOP10 (Invitrogen). From the resulting construct, the DNA fragment with promoter and CDS (coding DNA sequence) was restriction digested with SphI/ScaI and cloned into pAT18 [41] and resulted in pSARE-Red1. Transformants with pSARE-Red1 were selected on LB agar supplemented with 300 µg ml^−1^ of erythromycin.

### Coating of microtiter plates with ECM proteins

Preparation of 96-wells polystyrene microtitre plates (Falcon) surface-coated with ECM proteins was based on a previously described protocol [42]. The ECM proteins consisted of collagen I (Millipore, 08-115), III (Millipore, CC078) and IV (Sigma, C7521), laminin-α2 (Millipore, CC085), elastin (Sigma, E1625) and insoluble fibronectin (i-fibronectin, Sigma, F2518). BSA (bovine serum albumin, Sigma, A3803) was used as a control for specific adhesion to ECM proteins. Basically, ECM proteins were solubilised in 0.1 M carbonate coating buffer (pH 9.6) and 250 µl was dispensed at a saturating concentration (50 µg ml^−1^) to the well surface and incubated overnight at 4°C. The wells were washed with PBS (phosphate buffered saline, Sigma) containing 0.05% (v/v) Tween 20 (PBST, pH 7.3) at room temperature (rt) prior to blotting with 250 μl of 1% (w/v) BSA in PBST. After 2 h at 37°C, the wells were washed 3 times with PBST and used for bacterial adhesion or biofilm formation assays.

### Bacterial adhesion assay

Precultures were diluted 1∶100 and grown as described above. Relevant media were adjusted with NaOH (0.1 M) to reach a pH of 7 at the time of sampling. Sampling was performed during the exponential growth phase at an OD_600nm_ of 0.5, i.e. about 10^8^ CFU ml^−1^. Chloramphenicol was added and mixed gently at a final concentration of 90 µg ml^−1^ to prevent *de novo* protein synthesis and growth during the time of contact of bacterial cells with ECM proteins in the adhesion assay. Vigorous shaking, vortexing and centrifugation were avoided to preserve cell surface supramolecular structures potentially involved in adhesion. *E. coli* O157:H7 cell suspension (200 µl) was deposited in relevant protein-coated wells of the microtitre plate using wide-bore tips. Control wells were filled with sterile nutrient medium. Microtiter plates were incubated statically at relevant temperature for 2 h. After incubation, bacterial suspension was removed by pipetting. Wells were further first washed with TS (tryptone salt) to remove loosely attached cells. Adherent bacteria were fixed with 200 µl absolute ethanol for 20 min. Wells were then emptied by pipetting and dried for 30 min prior to 20 min staining with 200 µl of an aqueous-solution of crystal violet (0.1% w/v). Wells were emptied, washed a second time with TS to remove the excess of unbound crystal violet dye, and dried for 30 min. The bound dye was solubilized from stained cells using 200 µl of an aqueous solution of acetic acid (33% v/v) for 1 min under orbital shaking. Contents of each well (150 µl) were transferred to a clean microtiter plate and absorbance was measured at 595 nm using a microtiter plate reader. The readings were corrected by substracting the average absorbance from control wells.

To observe the influence of lower temperatures on initial adhesion of bacteria at pH 7, the microtiter plates were incubated statically at relevant temperature (39, 25, 7 and 4°C) for 2 h. To test the influence of subsequent exposure to lower temperatures upon initial adhesion, bacterial suspension was removed after 2 h of incubation at 25°C. Then, 200 µl of sterile medium was added to each well and were further incubated for 2 h at the relevant temperature.

To investigate the influence of pH, cacodylate buffers (0.5 M final) were used to adjust the pH of the bacterial culture samples at relevant pH (7.0, 6.5, 6.0 and 5.5), resulting in a 1:3 dilution of the bacterial culture sample. After inoculation, the microtiter plates were incubated statically at 25°C for 2 h. To test the influence of subsequent exposure to acidic pH upon initial adhesion, bacterial suspension was removed after 2 h of incubation at 25°C and pH 7. Then, 200 µl of sterile LB, adjusted at the relevant pH with 0.5 M final cacodylate buffers, were added and the microplates were further incubated for 2 h at 25°C.

### Autoaggregation assay

Precultures in LB, BJIC or DMEM were diluted 1∶100 and grown as described above. Based on a previously described assay [43], the cell suspension was adjusted to the same OD_600 nm_, chloramphenicol was added at a final concentration of 90 µg ml^−1^ and each culture was placed in conical tubes. No vigorous shaking, pipetting or vortexing was ever applied to preserve cell surface determinants potentially involved in autoaggregation. The tubes were incubated statically and vertically at 25°C. Samples of 500 µl were taken from the top of the tube at different time points to measure the OD_600 nm_. To confirm the autoaggregation, observations in phase-contrast microscopy were performed after 24 h of incubation.

### Biofilm formation assay

This assay was based on a previously described protocol [44]. For the starting inoculum, precultures in LB were diluted 1∶100 and 200 µl of bacterial cell suspension were dispatched in protein-coated wells of the microtitre plate and further incubated at 25°C statically. Control wells were filled with sterile nutrient medium. To test the influence of collagens in solution on biofilm formation, collagen I or III was added to the cell suspension prior to dispatching in the wells of uncoated microtitre plates and BSA was used as a control. To follow the bacterial sessile development at various incubation times, wells were subjected to the same sequential procedure described for the bacterial adhesion assay, i.e. washing with TS, fixation with absolute ethanol, air drying, staining with crystal violet solution, washing with TS, air drying, dye recovering in acetic acid solution and reading of the absorbance at 595 nm. To back up the results of the crystal violet assay, fluorescence microscopic observations were performed with the *E. coli* O157:H7 EDL933 *stx* ^−^ pSARE-Red1.

### Image acquisition and analysis from microscopic observations

Sample preparations were inserted on the stage plate to take one image for each well. This operation was repeated 12 times in order to acquire satisfactory statistical information. Field of view was chosen in the center of the well in order to avoid artifacts such as edge optical aberrations or biased bacterial spatial distribution. Fields of bacteria were imaged using a 60× magnification (40× objective with ×1.5 intermediate magnification).

Observations were performed in phase contrast transmitted light or fluorescence reflected light. The corresponding images were acquired using an inverted phase-contrast microscope (Olympus IMT-2) coupled to a cooled CCD camera (Olympus DP30BW) optimized for high sensitivity fluorescence work and driven by the CellA software v3.2 (Olympus France SAS, Rungis, France). Images were acquired with a 40x objective allowing phase contrast microscopy (Olympus LWD-CD-PLAN40FPL, 0.55, 160/1). The fluorescence light source was a mercury short arc lamp (HBO103W/2, OSRAM, Augsburg, Germany). Fluorescence acquisition was fitted with a “Cyanine 3” cube containing 540 nm exciter, 620 nm emitter, and 565 nm dichroic mirror (31002a filter cube, Chroma, Rockingham, VT, USA).

Images were processed with the public-domain image processing and analysis program ImageJ v1.43 (NIH-RSB, Bethesda, MD, USA) (http://rsb.info.nih.gov/nih-image/about.html). The pixels corresponding to bacteria were extracted by thresholding segmentation of the light gray levels. The relative area of bacteria was assessed by quantifying the number of these pixels in regard to the total number of pixels in the field of view.

### Statistical analysis

Statistical analysis was performed from Excel using XLSTAT v2009.3.02. Data of assays result from at least five independent experiments, i.e. five biological replicates. On the figures, error bars thus represent the standard deviation from five independent experiments. For each experiment, a value was calculated from the average of repetitions performed in triplicate at fewest. The mean values from the biological replicates were compared to the mean values obtained with BSA used as a control. Data were statistically analyzed following Student's t-test with differences considered significant (*p*<0.05, *), very significant (*p*<0.01, **), highly significant (*p*<0.001, ***) or very highly significant (*p*<0.0001, ****).

## Results

### Growth media influence bacterial adhesion to the main muscle ECM components

Different growth media were used to evaluate bacterial adhesion to the predominant fibrous proteins of the muscle ECM. Besides chemically defined or complex basal nutrient media generally used for growth of EHEC strains in laboratory, more specific undefined media close to the conditions encountered in the digestive tract of ruminants, i.e. before faecal contamination of meat products, were also used, namely LH (Leedle Hespell) containing fluid rumen content (22), BJIC (bovine jejunum-ileum content) and BCC (bovine caecum content) [38].

**Figure 1:**
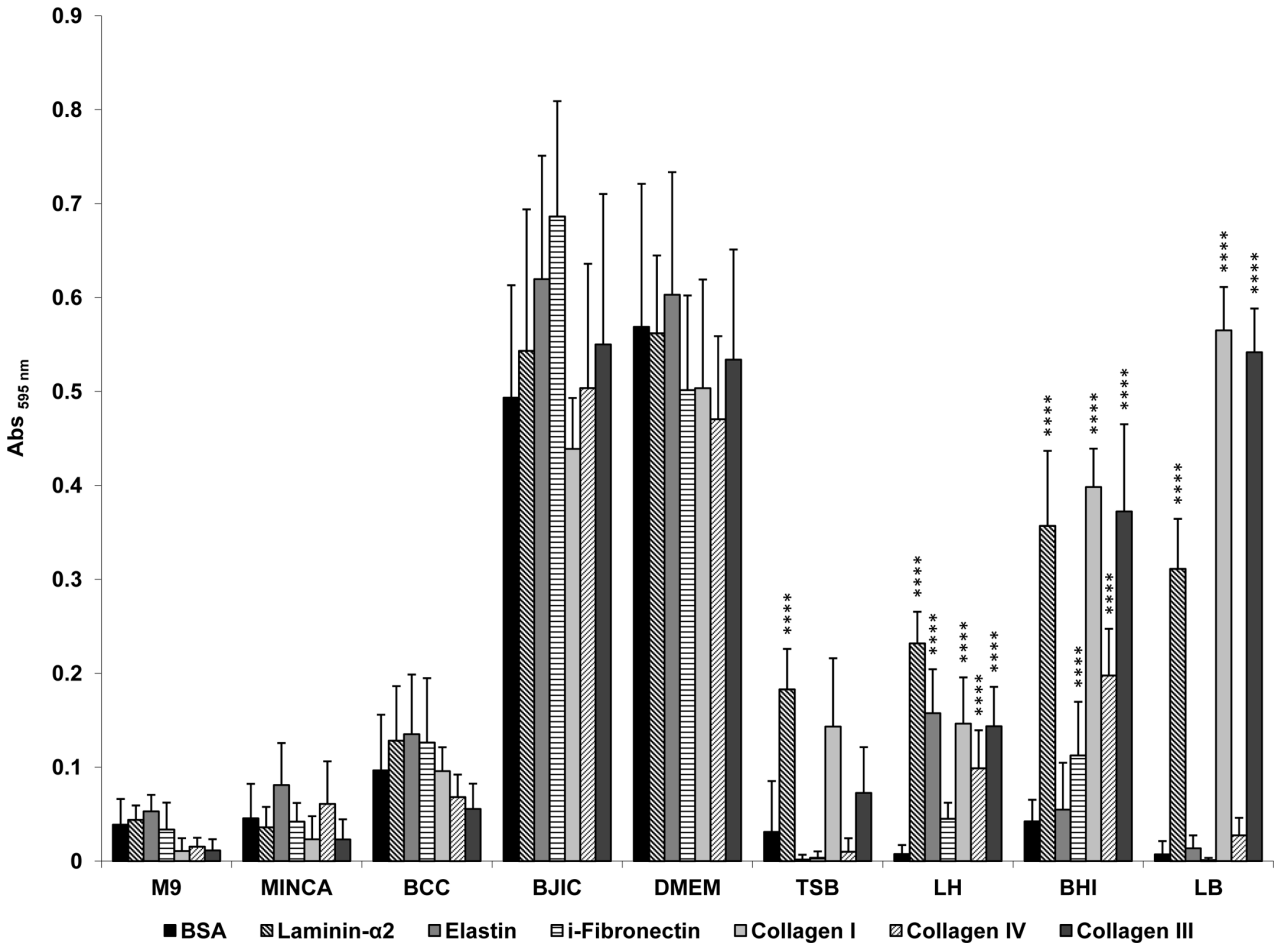
Adhesion to immobilized ECM proteins of *E. coli* O157:H7 EDL933 *stx* ^−^ grown in different media. Specific bacterial adhesion assay to the main ECM fibrous proteins present in meat was performed at 25°C using BSA as a control and measured by the crystal violet staining method. Bacterial cells for the adhesion assay were first grown at 39°C.

**Figure 2:**
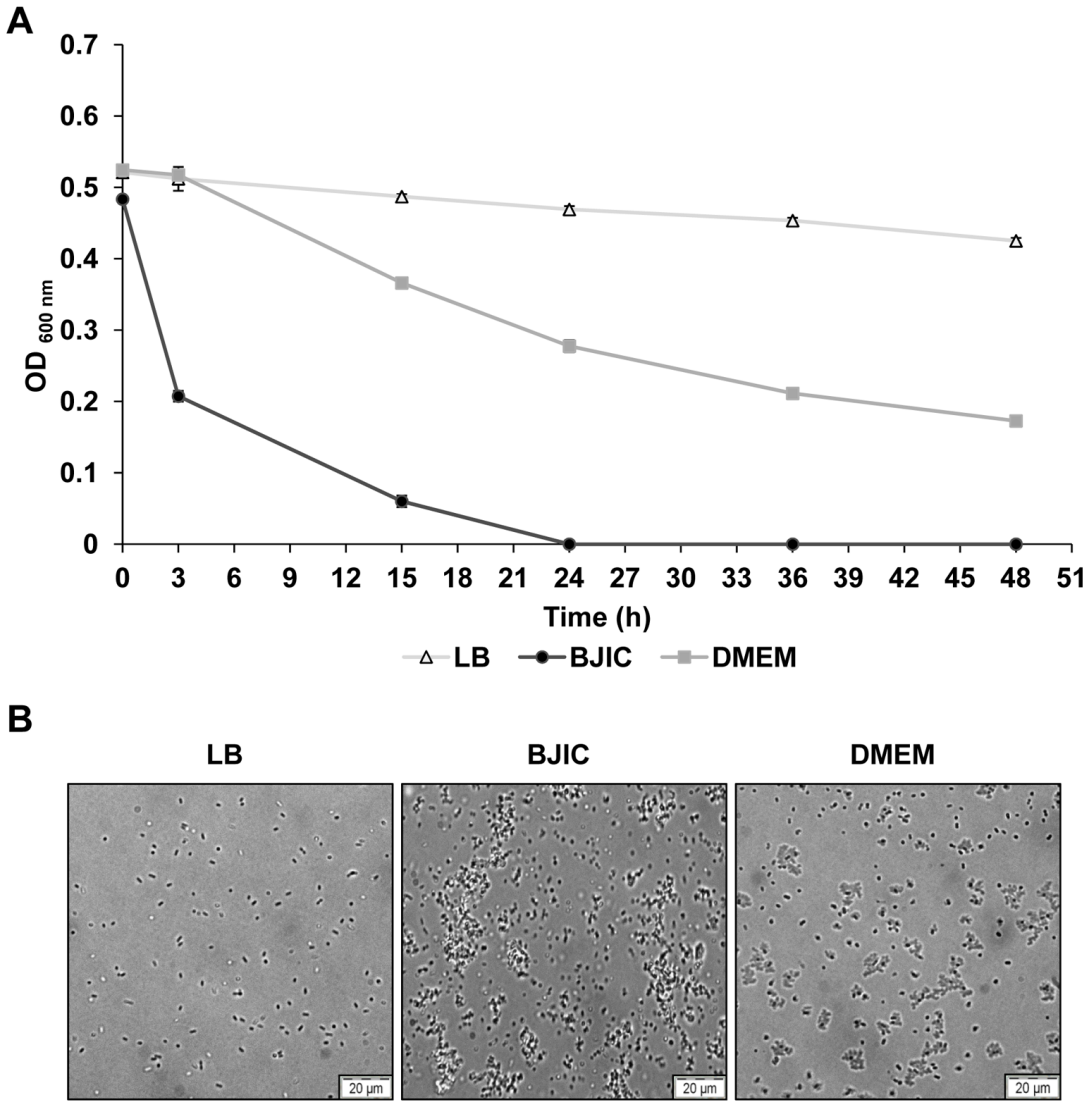
Autoaggregation of *E. coli* O157:H7 EDL933 *stx* ^−^. A: The autoaggregation assay was performed at 25°C on bacterial cells grown in DMEM or BJIC using LB as a control. B: The autoaggregation phenotype was visualized by phase-contrast microscopy following sampling at 24 h incubation time. Bacterial cells for the assay were first grown at 39°C.

It appeared that as for the control wells coated with BSA, *E. coli* O157:H7 EDL933 *stx* ^−^ grown in the chemically defined media M9 or MinCa could not adhere to the main muscle ECM components (Figure 1). In contrast, bacterial cells grown in BCC, BJIC or DMEM adhered similarly to the different ECM proteins tested as well as to BSA, indicating bacterial adhesion was non-specific. The levels of non-specific bacterial adhesion were especially high with BJIC and DMEM. As revealed by assays and miscroscopy observations (Figure 2), autoaggregation occurred in DMEM and BJIC but not in LB, where specific adhesion to some ECM components could be observed as described here below.

**Figure 3:**
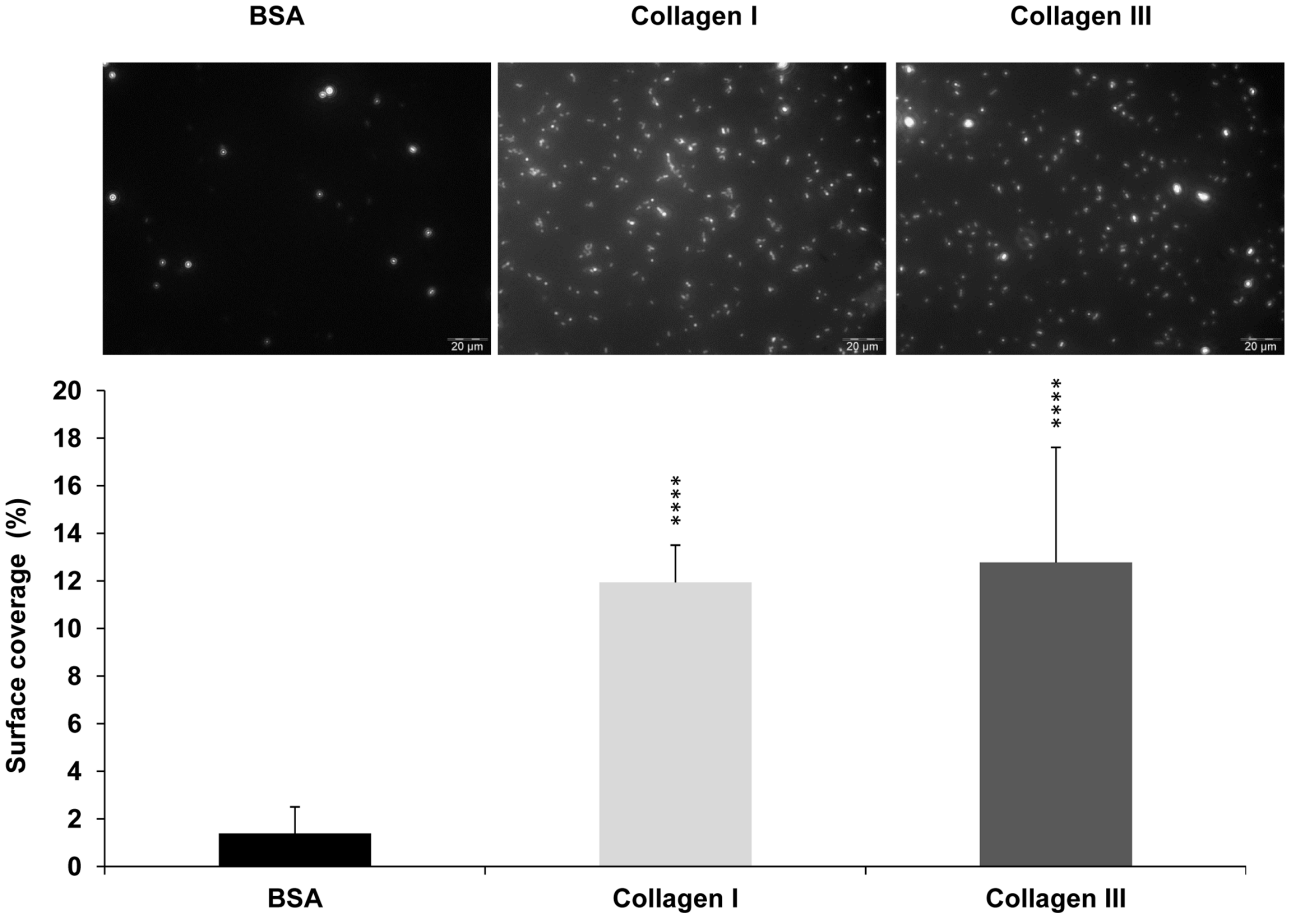
Adhesion to collagens I and III of *E. coli* O157:H7 EDL933 *stx* ^−^ strain grown in LB at 39°C. Epifluorescence microscopy data expressed as the percentage of area covered by fluorescent *E. coli* O157:H7 EDL933 *stx* ^−^ cells grown in LB in wells coated with collagen I or III with BSA used as control.

For the remaining media tested, specific adhesion of *E. coli* O157:H7 EDL933 *stx* ^−^ to ECM proteins could be observed (Figure 1). It clearly appeared that adhesion ability greatly depends on the growth media. Specific bacterial adhesion to laminin-α2 from cells grown in TSB, LH, BHI and LB could be observed as well as to elastin in LH or insoluble fibronectin (i-fibronectin) in BHI. Specific bacterial adhesion to both FFC (fibril-forming collagen), i.e. collagens I and III, and NFC (network forming collagen), i.e. collagen IV, [25] occurred with bacterial cells grown in LH or BHI. The most prominent specific adhesion of *E. coli* O157:H7 EDL933 *stx* ^−^ was observed against fibrillar collagen of type I or III with bacterial cells grown in LB. The similar trend of bacterial adhesion to immobilized collagen I or III versus BSA were further backed up by fluorescent microscopy observations using *E. coli* O157:H7 EDL933 *stx* ^−^ pSARE-Red1 (Figure 3).

### Temperature and pH influence bacterial adhesion to collagens I and III

Besides decrease of the meat temperature along the beef production chain (i.e. from 39°C, the temperature of the ruminant, work at room temperature until completion of slaughter, 7°C during transport, storage and/or further cutting, and 4°C during storage immediately after production), the decrease of pH (dropping from neutral to an ultimate value, i.e. pH_u_, of about 5.5) is one of the most prominent post-mortem physicochemical modifications occurring in skeletal muscles. Focusing on specific adhesion of *E. coli* O157:H7 EDL933 *stx* ^−^ grown in LB at 39°C to collagen I or III, the influence of the temperature and pH was investigated.

**Figure 4:**
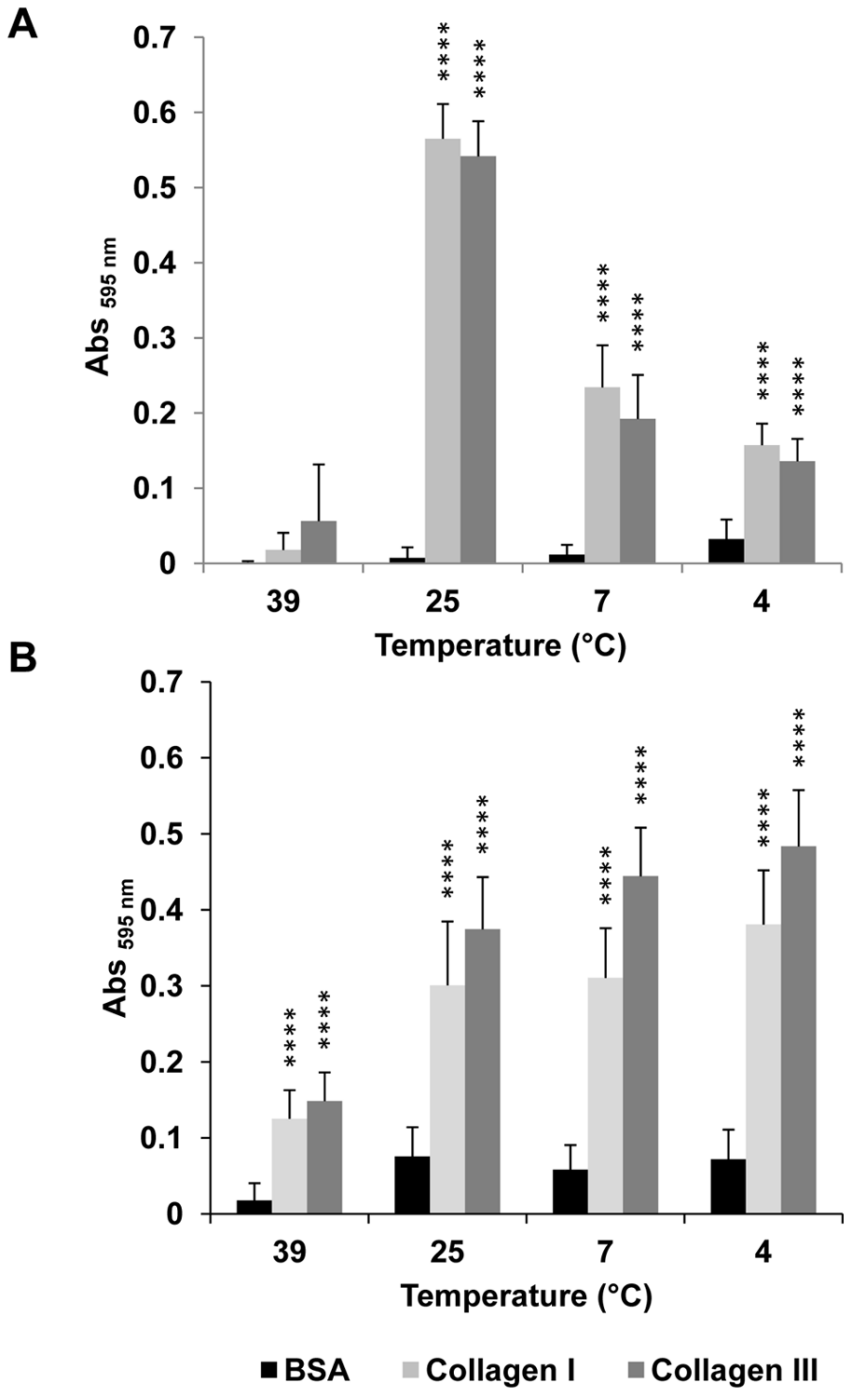
Effect of temperature on adhesion to immobilized collagen I or III of *E. coli* O157:H7 EDL933 *stx* ^−^. A: Bacterial adhesion was tested at decreasing temperatures encountered in meat along the production chain line, i.e. 39, 25, 7 and 4°C. Bacterial cells for the adhesion assay were first grown in LB at 39°C. B: The effect of temperature variation on bacteria initially adhered at 25°.

No significant adhesion could be observed at 39°C but maximum specific bacterial adhesion occurred at 25°C. At the lower temperatures 7 or 4°C, specific bacterial adhesion to collagen I or III could still be observed but the amount of adherent biomass was lower (Figure 4A). On bacterial cells initially adhered at 25°C, though, subsequent exposure at 39°C maintained specific adhesion to these two FFCs and the amount of adhering cells to collagen I or III was not significantly affected at the lower temperatures of 7 and 4°C (Figure 4B).

**Figure 5:**
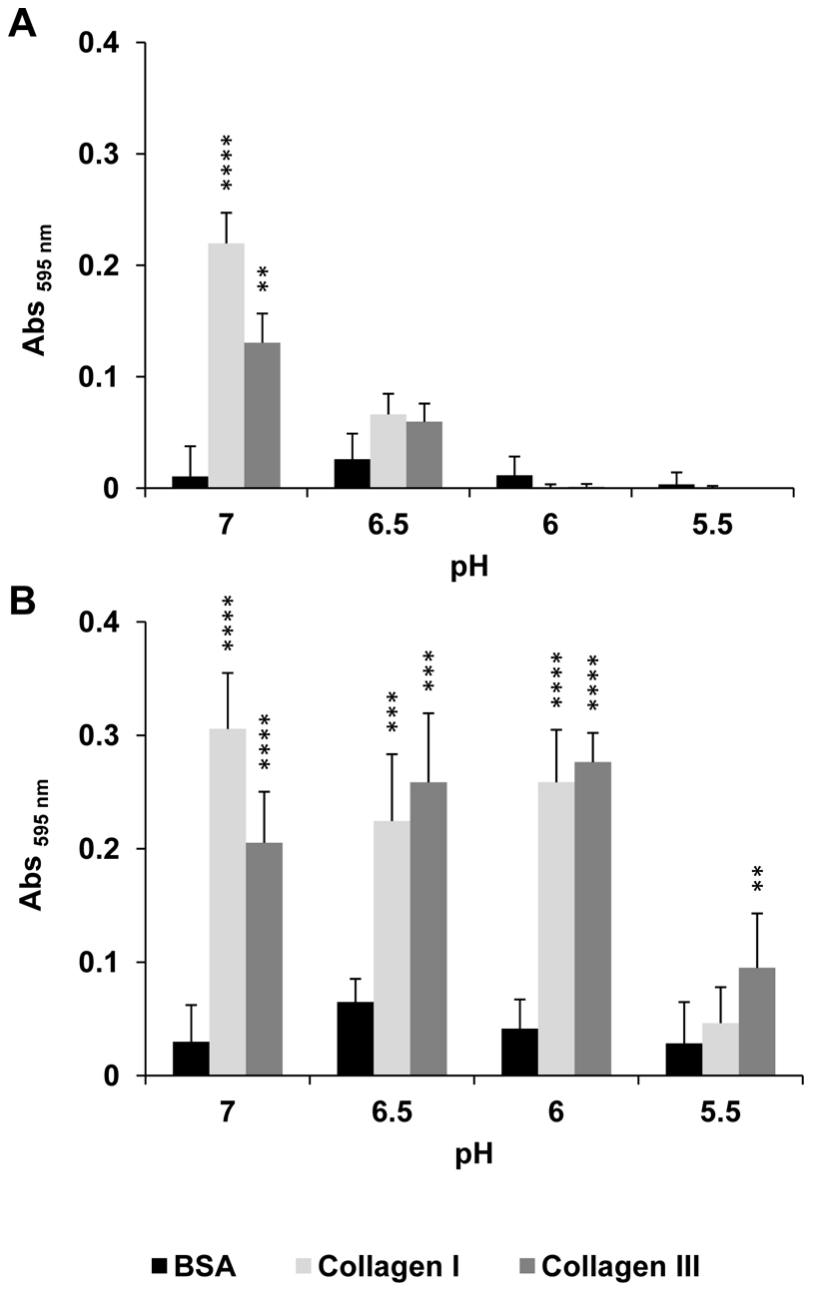
Effect of pH on adhesion to immobilized collagen I or III of *E. coli* O157:H7 EDL933 *stx* ^−^. A: Bacterial adhesion was tested at decreasing pH encountered post-mortem in skeletal muscle using cacodylate buffers to adjust the samples to the different pH. B: The effect of acidic pH on bacteria initially adhered at pH 7 was tested using sterile LB, which pH was adjusted with cacodylate buffers.

While these previous data were obtained at pH 7, the pH of the samples was adjusted with cacodylate buffers to investigate the influence of lower pH on specific bacterial adhesion at 25°C. It appeared that maximal bacterial adhesion occurred at pH 7 and chiefly decreased at lower pH where no significant specific bacterial adhesion could be observed (Figure 5A). On bacterial cells initially adhered at pH 7, though, subsequent exposure at lower pH did not significantly affect the amount of adhering cells to collagen I or III, except when exposed at pH 5.5 (Figure 5B). These results demonstrate the importance of temperature and pH conditions at the time of initial adhesion of *E. coli* O157:H7 EDL933 *stx* ^−^ as well as pH_u_ to limit bacterial adhesion.

### Collagens I and III promote biofilm formation

**Figure 6:**
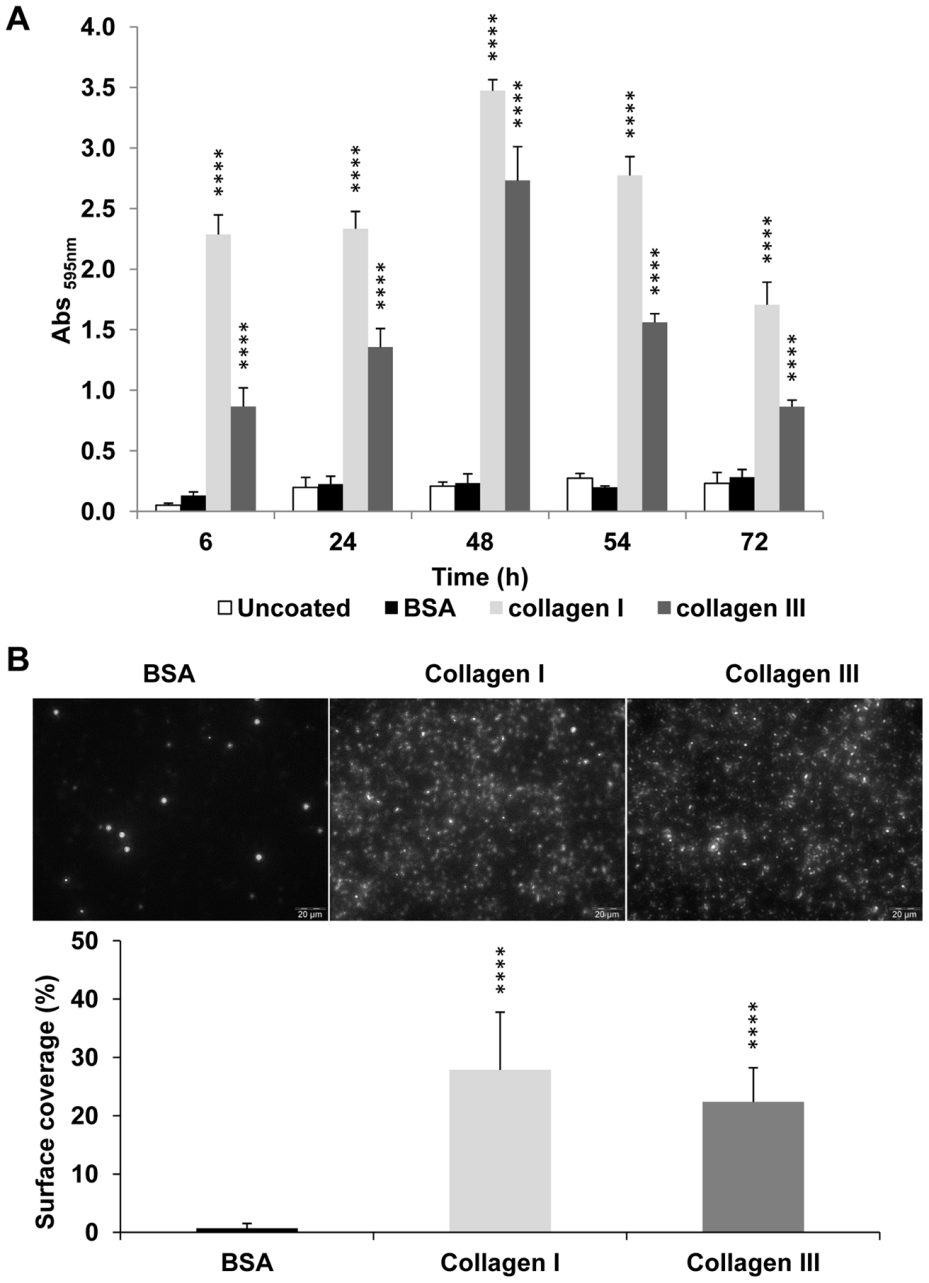
Colonization of immobilized collagen I and III by *E. coli* O157:H7 EDL933 *stx* ^−^. A: Kinetics of biofilm formation was performed in LB at 25°C using BSA as a control and measured by the crystal violet staining method. B: Percentage of surface coverage at 24 h from epifluorescence microscopy data of fluorescent *E. coli* O157:H7 EDL933 *stx* ^−^ pSARE-Red1 growing in LB at 25°C and colonizing surface with immobilized collagen I or III compared to immobilized BSA.

In addition to bacterial adhesion, the ability of *E. coli* O157:H7 EDL933 *stx* ^−^ to colonize immobilized collagen I or III was investigated. The presence of these coated ECM proteins chiefly increased the amount of sessile biomass over time when compared to uncoated surface or coated with BSA, especially immobilized collagen I (Figure 6A). In the course of sessile development on coated collagen, the sessile biomass increased up to 48 h of incubation before decreasing slowly as result of cell detachment upon washing following the crystal violet procedure. Observations in epifluorescence microscopy confirmed the increase of biofilm formation in the presence of immobilized collagen I or III (Figure 6B). There were very highly significant differences in biofilm formation on biotic surfaces made of immobilized BSA versus collagen I or III. The biofilm formation was clearly induced with coated collagen I or III.

**Figure 7:**
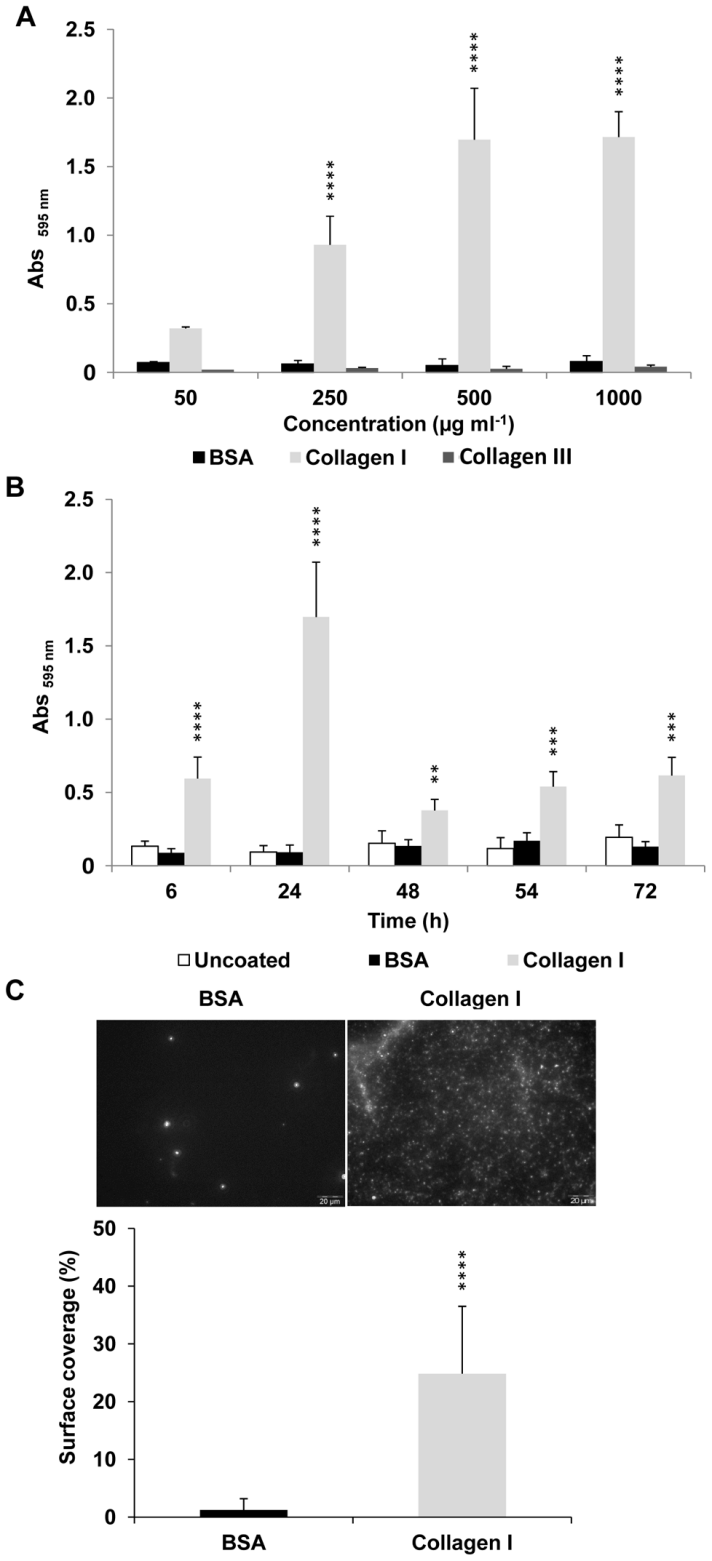
Surface colonization of *E. coli* O157:H7 EDL933 *stx* ^−^ with ECM proteins in solution. A: Surface colonization at 24 h of bacteria growing in LB at 25°C in presence of different concentration of collagen I or III in solution compared to BSA in solution and uncoated wells used as controls. B: Kinetics of biofilm formation in LB at 25°C with collagen I in solution (500 µg ml^−1^) compared to BSA in solution and uncoated wells used as controls. C: Percentage of surface coverage from epifluorescence microscopy data of fluorescent *E. coli* O157:H7 EDL933 *stx* ^−^ pSARE-Red1 growing in LB at 25°C and colonizing surface with collagen I in solution (500 µg ml^−1^) compared to BSA (500 µg ml^−1^) in solution.

To investigate the effect of ECM proteins in solution on bacterial surface colonization, different concentrations of collagen I or III were tested (Figure 7A). After 24 h growth, surface colonization was stimulated in a dose-dependent manner till 500 µg ml^−1^ of collagen I added to the culture medium but not by solubilized collagen III, which could not promote biofilm formation in this condition (Figure 7A). Following sessile development in the presence of 500 µg ml^−1^ of collagen I supplemented in the growth medium, the sessile biomass increased within the first 24 hours and decreased and stabilized from and after 48 h of incubation (Figure 7B). This decrease resulted from less adhering biomass. In the absence of collagen or BSA in solution, the amount of sessile biomass remained low and not significantly different from sessile development with coated BSA (Figures 7B and 6). Observations by epifluorescence microscopy confirmed that *E. coli* O157:H7 EDL933 *stx* ^−^ grown in LB supplemented with collagen I in solution, almost completely covered the surface but not with BSA in solution (Figure 7C).

## Discussion

When initial faecal contamination of beef carcasses occurs at dehiding stage, bacterial adhesion can occur on exposed skeletal muscles. The ECM in skeletal muscle is structured into the epimysium on the most outer layer, then the perimysium around the muscle fascicle and finally the endomysium around the single muscle cells, i.e. the muscle fibers [45]. In close proximity of the muscle fibers, the ECM forms the basal lamina (BL), of which the main fibrous ECM proteins are laminin-α2 (laminin-211 and -221, formerly called laminin-2 and -4, or M- and S-merosin, respectively) and network-forming collagen (NFC) of type IV, whereas the interstitial matrix(IM) is essentially composed of fibril-forming collagens (FFCs) of Type I and III as well as elastin [46], [47]. Insoluble-fibronectin (i-fibronectin) is present in these two forms of ECM (i.e. IM and BL). This knowledge of ECM organization at supramolecular, cellular, tissue and organ levels was here carefully considered for investigating adhesion of EHEC to the ECM [25]. In some previous reports on adhesion of EHEC to ECM muscle proteins, collagen III was overlooked and soluble fibronectin (s-fibronectin) or laminin-α1 (laminin-111, formerly called laminin-1) [26], [27], [31] were used although not relevant to skeletal muscle tissue.

This investigation pinpoints a crucial aspect quite overlooked in the literature [31], [48], that is the great influence of growth media on subsequent EHEC adhesion to ECM proteins. This point, however, could be expected from the numerous investigations reporting differential expression of virulence factors, including adhesins, depending on environmental conditions [30], [49], [50], [51], [52], [53], [54], [55], [56], [57], [58], [59]. Besides the usual laboratory nutrient growth media, more specific undefined media from intestinal contents of different parts of bovine gastro-intestinal tract (rumen, jejunum-ileum, caecum) were used for the first time to investigate bacterial adhesion in an attempt to mimic physiological conditions of the bovine intestine. In BJIC, non-specific bacterial adhesion to ECM proteins was observed and related with cell autoaggregation (similar result was observed for the chemically defined medium DMEM). This phenomenon could hinder specific bacterial adhesion to ECM proteins by some MSCRAMM (microbial surface components recognizing adhesive matrix molecules) proteins. In EHEC O157:H7 EDL933, at least one surface molecular determinant has been reported as potentially involved in such a phenotype. Indeed, its genome encodes an autotransporter characterized as a calcium-binding protein involved in autoaggregation and biofilm formation, namely Cah (calcium-binding antigen-43 homologue) [60],[61],[62]. Transcriptional gene reporter assays following fusion with the promoter of the *cah* gene indicated high expression level in DMEM over LB. However, genetic/protein expression and functional implication of *cah* in the autoaggregation phenotype has never been ascertained in EHEC O157:H7 EDL933, which would require further investigations in the light of our present findings.

While no specific ECM adhesion could be observed from bacterial cells in BCC, specific adhesion to laminin-α2, elastin, collagen I, IV and III occurred when grown in LH, a medium containing fluid ruminal content. This is especially relevant since this medium mimics an environment encountered by bacteria before shedding and subsequent faecal contamination of carcasses [63],[64]. Among the lab growth media, the foremost specific ECM-protein adhesion was here observed with bacterial cells grown in LB and against collagen I or III. This result indicates that specific molecular factor(s) for adhesion to the main muscle FFCs are expressed in these experimental conditions. In all tested conditions, specific adhesion to the main fibrous proteins found in the basal lamina of skeletal muscle tissue, i.e. NFC IV and laminin-α2, is much weaker. Altogether, this finding somehow contrasts with previous reports in the literature where no adhesion to collagen I but adhesion to collagen IV and laminin-α1 was reported for *E. coli* O157:H7 EDL933 [31],[48]. Besides environmental conditions (growth medium, temperature, pH or type of ECM proteins) [65],[66], it should be stressed that, as detailed in the Material & Methods sections, great care was here taken for handling the bacterial samples and preserving as much as possible cell-surface molecular determinants potentially involved in adhesion to ECM proteins. This concern is not trivial and should be thoughtfully considered in future studies of specific bacterial adhesion to ECM proteins. In addition, our study provides strong basements for further investigation of expression and functional analysis of MSCRAMM proteins involved in adhesion of EHEC O157:H7 EDL933 to ECM [25].

Surprisingly enough, bacterial adhesion to immobilized ECM components has been observed only once in EHEC O157:H7 [67]. However, they were not all relevant to skeletal muscle tissue. Indeed, only soluble fibronectin (and different proteolytic fragments), laminin-α1 and collagen IV were considered. Two major types of molecular mechanisms could explain bacterial adhesion to ECM components [13], i) outer membrane proteins, namely autotransporters (e.g. EhaA, EhaB, EhaG), and ii) cell-surface appendages, especially pili (e.g. HCP, Lpf). Upon cloning and expression of the individual autotransporters in *E. coli* K12 derivatives, specific adhesion to different ECM components was demonstrated [48],[68],[69],[70]. However, their implication has never been ascertained in an EHEC background because no adhesion was observed [48]. Using purified proteins, the affinity of pili for ECM components was investigated by plasmon resonance biosensor, flow cytometry or ELISA-based binding assay [26],[67],[71]. Of note, the binding of ECM components to EHEC was investigated using free forms and not immobilized forms of the ECM components, which is far different from a bacterial adhesion assay. Importantly, the different autotransporters and pili can each binds several different ECM components. In other words, adhesion of bacterial cells to ECM components is highly complex because it is multi-factorial and compensatory. While the use of LH, BJIC and BCC is highly biologically relevant, those media are also valued because of the difficulty to obtain the different contents of a bovine digestive tract. In the present investigation, the choice was then to use a common laboratory medium showing some similarities in the trend of bacterial adhesion to ECM components in LH. We also decided to focus on the most prominent ECM components enabling high specific bacterial adhesion, namely collagen I and III. Undoubtedly, much further in-depth investigations are necessary to elucidate the gene regulations and other molecular mechanisms responsible for the different patterns of bacterial adhesion to the different ECM components as observed in the different growth media.

Besides growth media, bacterial adhesion capacity is greatly influenced by the temperature and pH. While this suggests the affinity of the MSCRAMM protein(s) towards ECM protein is modified by such physicochemical parameters, it also provides some new research directions for the development of innovative practices and/or prevention strategies at critical points of the slaughter of animals in order to minimize the risk of contamination by EHEC O157:H7. Indeed, it pinpoints that maximal bacterial adhesion to the main FFCs present in skeletal muscle, which are present on surface of carcasses, occurs at a standard room temperature of 25°C, which can be attained in meat during the time of slaughter and initial cutting into quarters. In addition, maximum specific adhesion to collagen I or III occurred at pH 7, which suggests the critical point for contamination occurs at the beginning of slaughter. Moreover, once bacteria adhered, it appeared that further modifications of temperature or pH had few effects on specific adhesion of EHEC to collagen I or III, except at pH_u_.

In addition to specific adhesion, the presence of immobilized FFCs could promote the formation of dense biofilm. The induction of a dense biofilm was also observed in the presence of collagen I in solution but not with collagen III. It may be that collagen I in solution promotes aggregation, which in turn leads to flocculation and formation of a biofilm. While both collagens I and III are FFCs with the same supramolecular structure, they present differences in their composing subunits [25],[72]. In *Streptococcus suis*, the influence of soluble versus immobilized fibrinogen was similarly tested and reported the induction of biofilm formation only in the presence of the soluble fibrinogen [73]; Still, the molecular basis for this difference in bacteria – protein interactions depending on the solubilized or immobilized form of the ECM proteins remain to be elucidated [74],[75]. For EHEC, this constitutes a critical point for the control of bacterial contamination in the food plant environment considering residual collagen could be found solubilized in some fluids (meat juice, blood) resulting from meat processing (*e.g.* cutting, grounding) and/or immobilized (following soiling for instance) on sterilized workbenches or utensils, such as butcher knives.

Bearing in mind the main fibrous ECM proteins, especially collagen, are quite ubiquitous in animal tissues, bacterial cell attachment is certainly an adaptive strategy the bacterial species have evolved with in the course of interactions with eukaryotic cell [25],[76]. While this study focused on bacteria-protein interactions, our future investigations on EHEC adhesion/colonization of muscle fibers and meat will complete information regarding the interactions at molecular, cellular, ultrastructural and organ levels. A better understanding of the molecular and cellular mechanisms involved in EHEC adhesion to meat is a prerequisite to limit the risk of food poisoning by EHEC, whose outcomes can be serious complications or even life-threatening pathologies especially for young children [7]. Eventually, results of this research could lead to more effective and innovative prevention strategies in the meat industry to further limit or avoid carcass contamination by bacterial pathogens. They also provide further insight in the physiopathology of EHEC with an integrated view from the reservoir, the contaminated food products to the subsequent ingestion resulting in host infection.
